# Efficacy of Intermittent, Oral Famciclovir Prophylaxis for Bortezomib-Induced Herpes Zoster in Multiple Myeloma Patients

**DOI:** 10.3389/fonc.2022.843032

**Published:** 2022-03-11

**Authors:** Gaofeng Zheng, Fangshu Guan, Xiaoyan Han, Li Yang, Yi Zhao, Yang Yang, Enfang Zhang, Jingsong He, Donghua He, Wenjun Wu, He Huang, Zhen Cai

**Affiliations:** Multiple Myeloma Treatment Center and Bone Marrow Transplantation Center, The First Affiliated Hospital, School of Medicine, Zhejiang University, Hangzhou, China

**Keywords:** intermittent famciclovir, prophylaxis, herpes zoster, multiple myeloma, bortezomib

## Abstract

**Objective:**

To explore the efficacy and safety of intermittent, oral famciclovir prophylaxis for bortezomib-induced herpes zoster in multiple myeloma patients.

**Method:**

We retrospectively analyzed the incidence of bortezomib treatment-related varicella-zoster virus reactivation in 719 newly-diagnosed multiple myeloma patients receiving intermittent oral famciclovir prophylaxis, continuous oral acyclovir prophylaxis or no prophylaxis. The definition of intermittent oral famciclovir prophylaxis was oral famciclovir at a dose of 250mg twice daily for 9 days after finishing the last dose of bortezomib therapy every cycle. Age, gender, stage per the International Staging System, type of M protein, baseline of absolute lymphocyte count, absolute neutrophil count, and absolute monocyte count were analyzed to find the potential factors that could predispose to herpes zoster infections.

**Results:**

Varicella-zoster virus infection occurred in 96 patients (13.4%) during bortezomib treatment. The incidence of herpes zoster was significantly higher in the non-prophylaxis group compared with the prophylaxis group (22.9% vs 8.2% P<0.001), while the rate was similar between the intermittent oral famciclovir group and the continuous oral acyclovir group (8.4% vs 7.9% P=0.835). Hepatic and renal toxicity were observed in 12% and 2.8% of the patient respectively in the intermittent famciclovir group, which was similar in the continuous acyclovir group (18.1% and 4.2%). The prophylactic use of antiviral agents is a predictive factor for varicella-zoster virus reactivation.

**Conclusion:**

Intermittent famciclovir prophylaxis is effective and safe in preventing herpes zoster development and can markedly reduce the duration of oral medicine treatment compared with continuous acyclovir prophylaxis.

## Introduction

Multiple myeloma (MM), a malignant neoplasm of plasma cells in bone marrow, is the second most common hematologic malignancy worldwide. Bortezomib, as a backbone therapy for MM, is effective for the treatment of myeloma patients and contributes to an improved response rate and survival. However, one of the adverse effects emerged was notably the reactivation of latent varicella-zoster virus (VZV) infections. In the APEX study, bortezomib cohort showed a 13% rate of herpes zoster (HZ) infection, compared to 5% in the dexamethasone cohort only (1). Other studies have also reported an increased occurrence of VZV reactivation, ranging from 9.2%-22.3% to even as high as 60% (2, 3). As such, the National Comprehensive Cancer Network Guidelines for Multiple Myeloma recommend antiviral prophylaxis for all patients treated with proteasome inhibitors (4). Yet, the explicit prevention programs have not been established.

Previous studies have suggested that the continuous use of acyclovir could effectively suppress VZV reactivation (5)—the dose of acyclovir being from 200mg once daily to 400mg three times a day (6), a dosage which is also widely used in real-world clinical work. However, the long-term use of acyclovir may cause an increased incidence of adverse events—especially renal and neurological toxicity, affecting the quality of daily life and creating a psychological burden for patients (7). Thus, there is a rationale for exploring a more reasonable prophylaxis scheme. In this report, we retrospectively analyzed the efficacy and safety of intermittent oral famciclovir prophylaxis for bortezomib-induced herpes zoster in MM patients.

## Materials and Methods

### Patients

We reviewed the records of all MM patients who had been treated in our hospital between January 2010 to November 2019. In total, 719 newly diagnosed MM patients who received at least two cycles of bortezomib-based therapy with proper follow-up were included in this study. The diagnosis of MM was in accordance with the International Myeloma Working Group criteria. We used the International Staging System (ISS) staging in assessing patients. This study protocol was approved by the Research Ethics Committee of the First Affiliated Hospital, College of Medicine, Zhejiang University.

### Treatment

Patients received bortezomib-based combination therapy, including bortezomib plus dexamethasone (VD) and triplet combinations of VD with adriamycin (VAD), cyclophosphamide (VCD), thalidomide (VTD), lenalidomide (VRD) and melphalan (VMP). In these regimens, bortezomib was either given on days 1, 4, 8, and 11 every 28 days or on days 1, 8, 15, and 22 every 35 days. The standard dose was 1.3mg/m^2^, and it was reduced to 1.0mg/m^2^ or 0.7mg/m^2^ at the occurrence of peripheral neuropathy according to the recommend dose-modification guidelines (8).

### Study Design

Patients were divided into three groups according to the different prophylactic regimens. The non-prophylaxis group was defined as not being administered any antiviral prophylaxis or intravenous immune globulin during the entire period of bortezomib therapy. The continuous acyclovir group was continuously taking acyclovir with a dose of 200mg three times daily (the dose was adjusted according to creatinine clearance (Ccr): Ccr below 25ml/min, acyclovir with a dose of 100mg three times daily) during the entire bortezomib therapy. The definition of the intermittent famciclovir group was that they were receiving oral famciclovir with a dose of 250mg twice daily (the dose was adjusted according to Ccr: Ccr between 20-39ml/min, famciclovir with a dose of 250mg once daily, Ccr below 20ml/min, famciclovir with a dose of 125mg every 2 days) for 9 days after finishing the last dose of bortezomib therapy every cycle. The definition and analysis of herpes zoster infection was confirmed using the National Cancer Institute’s Common Terminology Criteria for Adverse Events version 5.0.

### Statistical Analysis

The software *SPSS 26.0* was used for data analysis. Patients and disease-related factors between the different groups were compared using the chi-squared test for categorical variables, analysis of variance, and the Kruskal-Wallis test for continuous variables. The logistic regression model was used to assess potential factors that could predispose to herpes zoster infections. All probability values were two-sided and a P-value of 0.05 indicated statistical significance.

## Results

### Patient Characteristics

A total of 719 patients were enrolled in this retrospective cohort study, the median age was 62 years (rang, 27-85). Of these, 253 patients had no prophylaxis, 216 patients had continuous acyclovir prophylaxis, and 250 patients had intermittent famciclovir prophylaxis. The baseline demographic and disease characteristics are listed in **Table 1**. There were no significant differences in age, gender, ISS stage, and the median hematologic parameters at the baseline among the three cohorts – but the immunoglobulin (Ig) types varied. The rate of IgG type was higher, and the rate of the light chain was lower in the intermittent group. Patients in the intermittent or continuous group received more cycles of bortezomib therapy than those in the non-prophylaxis group, the median cycles were 7(2-18), 8(2-18) and 4(2-15), respectively. The combination treatment regimens also varied.

**Table 1:** The demographic and baseline clinical characteristics.

	Total (N=719)	No prophylaxis group (n=253)	Continuous acyclovir group (n=216)	Intermittent famciclovir group (n=250)	P value
Median age, years (range)	62 (27-85)	61 (27-84)	63 (39-85)	62 (31-84)	0.181
Male, n (%)	413 (57.4)	153 (60.5)	122 (56.5)	138 (55.2)	0.461
ISS stage, n (%)					0.105
I	209 (29.1)	64 (25.3)	58 (26.9)	87 (34.8)	
II	232 (32.3)	83 (32.8)	78 (36.1)	71 (28.4)	
III	278 (38.7)	106 (41.9)	80 (37)	92 (36.8)	
Myeloma types, n (%)					0.038
IgG	317 (44.1)	103 (40.7)	113 (52.3)	101 (40.4)	
IgA	189 (26.3)	64 (25.3)	54 (25)	71 (28.4)	
IgD	19 (2.6)	6 (2.4)	4 (1.9)	9 (3.6)	
IgM	2 (0.3)	0 (0)	2 (0.9)	0 (0)	
Biclonal (IgA、IgG)	1 (0.1)	0 (0)	0 (0)	1 (0.4)	
Light chain	175 (24.3)	76 (30%)	39 (18.1)	60 (24)	
Non secretory	16 (2.2)	4 (1.6)	4 (1.9)	8 (3.2)	
Hematologic parameters at baseline, median (range)					
White blood count	4.9 (1.6-21.4)	5 (1.6-12.9)	4.8 (1.8-16.3)	5 (2.4-21.4)	0.162
Absolute neutrophil count	2.8 (0.3-13.1)	2.82 (0.5-11.5)	2.65 (0.3-9.5)	2.9 (1.1-13.1)	0.132
Absolute lymphocyte count	1.47 (0.2-10.4)	1.4 (0.2-4.9)	1.455 (0.5-4)	1.49 (0.2-10.4)	0.804
Absolute monocyte count	0.37 (0-4.9)	0.4 (0.02-4.9)	0.38 (0.03-3.68)	0.33 (0-3.9)	0.15
Treatment regimen, n (%)					<0.001
VD	90 (12.5)	35 (13.8)	23 (10.6)	32 (12.8)	
VAD	121 (16.8)	49 (19.4)	40 (18.5)	32 (12.8)	
VCD	423 (58.8)	135 (53.4)	113 (52.3)	175 (70.0)	
VTD	28 (3.9)	13 (5.1)	14 (6.5)	1 (0.4)	
VRD	32 (4.5)	10 (4.0)	16 (7.4)	6 (2.4)	
VMP	13 (1.8)	0 (0.0)	10 (4.7)	3 (1.2)	
Others	12 (1.7)	11 (4.3)	0 (0.0)	1 (0.4)	
Median number of treatment courses, median (range)	6 (2-18)	4 (2-15)	8 (2-18)	7 (2-18)	<0.001

### Varicella-Zoster Virus Reactivation

VZV reactivation occurred in 96 patients (13.4%) during bortezomib treatment. Among those, 58 cases were in the non-prophylaxis group, 17 were in the continuous group, and 21 were in the intermittent group. The incidence of HZ was significantly higher in the non-prophylaxis group compared with the prophylaxis group (22.9% vs 8.2% P<0.001). By contrast, the rate was similar between the intermittent group and the continuous group (8.4% vs 7.9% P=0.835).

The median dosage of bortezomib was 8 (1-28) when the patients developed VZV reactivation, and the cumulative dose was 16 (2.2-56). 39 patients had grade 1 or grade 2 HZ, and the other 57 patients had grade 3 HZ. No grade 4 or grade 5 HZ was observed. In the non-prophylaxis group, more patients had grade 3 HZ (70.7%, P=0.038) (**Table 2**). All the patients presented with localized infection and were cured after medical treatment.

**Table 2:** Characters of VZV reactivation.

	Total (N=719)	No prophylaxis group (n=253)	Continuous acyclovir group (n=216)	Intermittent famciclovir group (n=250)	P value
Incidence of HZ infection, n (%)	96 (13.4)	58 (22.9)	17 (7.9)	21 (8.4)	<0.001
Median dose of bortezomib therapy, median (range)	8 (1-28)	8 (2-28)	8 (3-24)	8 (1-24)	0.326
Cumulative bortezomib dose, median (range)	16 (2.2-56)	13.8 (4-56)	15.2 (8.37-54.4)	18 (2.2-52.9)	0.194
Grade of infection					0.038
1-2	39 (40.6)	17 (29.3)	9 (52.9)	13 (61.9)	
3	57 (59.4)	41 (70.7)	8 (47.1)	8 (38.1)	
Postherpetic neuralgia, n (%)	22 (22.9)	12 (20.7)	4 (23.5)	6 (28.6)	0.703

Fifty-five patients delayed bortezomib treatment due to HZ infection. The median delayed period was 12 days (range, 4-180 days). 9 patients discontinued bortezomib therapy because of disease progression or the patients’ refusal of any further bortezomib treatment due to fear of the recurrence of shingles. The treatment schedule of the other 32 patients was not affected. 22 patients developed postherpetic neuralgia, 1 of whom had to receive nerve block operation to relieve the pain.

### Hepatic and Renal Toxicity

Hepatic and renal toxicities (**Table 3**) during the entire treatment were comparable in the continuous acyclovir and intermittent famciclovir groups. Liver toxicity (alanine aminotransferase and/or aspartate aminotransferase levels above the upper limit of normal) was reported in 18.1% of patients in the continuous acyclovir group and in 12% of patients in the intermittent famciclovir group(P=0.066). Only 5 patients experienced grade 2-3 liver toxicity (2 patients in the continuous acyclovir group and 3 patients in the intermittent famciclovir group). Within the study group as a whole, increases in creatinine occurred in 9 patients in the continuous acyclovir group and in 7 patients in the intermittent famciclovir group, respectively (P=0.721). For those patients with baseline renal impairment, only 4 patients (4/69) had further-elevated creatinine levels in the continuous acyclovir group and only 3 patients (3/79) in the intermittent famciclovir group. No patients had a persistent elevation of the creatinine and aminotransferase level.

**Table 3:** Hepatic and renal toxicities.

	Intermittent famciclovir group (n=250)	Continuous acyclovir group (n=216)	P value
Hepatic toxicity, n (%)			0.066
Grade 1	27 (10.8)	37 (17.1)	
Grade 2	2 (0.8)	1 (0.46)	
Grade 3	1 (0.4)	1 (0.46)	
Renal toxicity, n (%)	7 (2.8)	9 (4.2)	
Increased serum creatinine level at baseline, n (%)	79 (31.6)	69 (31.9)	

### Predictive Factors for Herpes Zoster Infection

Univariate analysis (**Table 4**) shows that HZ infection is strongly associated with prophylaxis (P<0.001) - the proportion of patients with no prophylaxis was higher in the HZ infection group. Other factors such as gender, age, ISS stage, type of M protein, baseline of ALC, ANC and AMC had no relationship with VZV reactivation.

**Table 4:** Predictive factor for HZ infection.

	HZ infection (n=96)	Without HZ infection (n=623)	Univariate analysis P value
Sex, male, n (%)	56 (58.3)	357 (57.3)	0.849
Age, years (range)	63 (47-80)	61 (27-85)	0.053
ISS stage, n			0.506
1	31	178	
2	33	199	
3	32	246	
Baseline WBC, ×10^9^/L	4.8 (2.5-12.9)	4.9 (1.6-21.4)	0.762
Baseline ANC, ×10^9^/L	2.7 (0.4-11.5)	2.8 (0.3-13.1)	0.817
Baseline ALC, ×10^9^/L	1.5 (0.5-4.9)	1.45 (0.2-10.4)	0.714
Baseline AMC, ×10^9^/L	0.4 (0.08-3.9)	0.36 (0-4.9)	0.287
Myeloma types, n (%)			0.838
IgG	40 (41.7)	277 (44.5)	
IgA	24 (25)	165 (26.5)	
IgD	3 (3.1)	16 (2.6)	
IgM	0 (0)	2 (0.3)	
Biclonal (IgA、IgG)	0 (0)	1 (0.2)	
Light chain only	28 (29.2)	147 (23.6)	
Non secretory	1 (1.0)	15 (2.4)	
Prophylaxis, n			<0.001
No	58	195	
Continuous acyclovir	17	199	
Intermittent famciclovir	21	229	

## Discussion

In this retrospective, single-center cohort study, different prophylactic regimens to prevent bortezomib-related herpes zoster in MM patients were analyzed to find an optimal prophylaxis. This is an ample-sample research on comparing the efficacy of intermittent and continuous prophylaxis. As we know, famciclovir, the oral prodrug of penciclovir, has higher bioavailability than acyclovir, and its half-life is also longer than that of acyclovir, which requires fewer tablets and less-frequent administration when compared to acyclovir (9). Thus, in our clinical practice, we empirically used intermittent oral famciclovir to prevent HZ infection at a dose of 250mg twice-daily after finishing the last dose of bortezomib therapy every cycle. In the end, 250 patients with intermittent prophylaxis and 719 patients in total who were treated from January 2010 to November 2019 were included in this study. We observed that intermittent use of famciclovir was effective and safe in reducing the incidence of herpes zoster events.

Prior research has proven that the prophylactic use of antiviral drugs can significantly reduce the incidence of bortezomib-related shingles. A Spanish clinical trial demonstrated that the incidence of herpes zoster infection can be decreased from 13% to 6.6% after receiving acyclovir 400 mg three times daily as an acyclovir prophylaxis (10). Several other clinical trials have adopted the dosage of 400 mg three times daily for prophylaxis (6). Another study showed that a daily dosage of 500 mg valacyclovir is also beneficial for preventing viral reactivation (11). In an attempt to reduce the potential side-effects caused by prophylaxis, low-dose acyclovir has been used to prevent VZV reactivation. Kim et al. found that a daily 400mg of acyclovir was effective for the prevention of herpes zoster (12), while Minarik et al. showed that 200 mg acyclovir was sufficient (13).

However, in most of these studies antiviral agents were continuously administered. The long term use of antiviral drugs may induce nephrotoxicity and poor therapeutic compliance. A small-sample study from Shanghai retrospectively analyzed the incidence of HZ between the continuous and intermittent-administration groups and demonstrated the intermittent usage of valacyclovir an effective and safe prophylaxis. They also found that 2 patients in the continuous administration group receiving prophylaxis might aggravate anxiety and depression (14).

In our research, we reported a 13.4% rate of VZV reactivation, which is similar to previous studies. The rates of VZV reactivation were 8.4% (21/250) in the intermittent group, 7.9% (17/216) in the continuous group, and 22.9% (58/253) in the non-prophylaxis group, respectively. Furthermore, the severity of herpes was similar between the intermittent and continuous groups, while patients in non-prophylaxis group had more grade-3 HZ (70.8%). So we demonstrated that prophylaxis is beneficial for suppressing VZV reactivation, and intermittent famciclovir achieved equal efficacy to continuous acyclovir in preventing herpes zoster development. In addition, intermittent prophylactic measures can clearly reduce the days of medication and frequency of administration, which allows prolonging of drug-free time and improving treatment compliance in patients.

A common complication of shingles is post-herpetic neuralgia, which seriously affects the patients’ quality of life (15). Serious infectious events may hinder the continuation of anti-myeloma therapy and increase hospitalization days. In our study, 22 patients suffered from postherpetic neuralgia, and the treatment plan of 64 patients was affected. The incidence of postherpetic neuralgia had no difference between the three groups.

As we can see, the baseline characteristics including age, gender, ISS stage, and the median hematologic parameters at the baseline among the three cohorts were similar. But median cycles of treatment were less in non-prophylaxis cohort. The main reason was that the diagnosed date was earlier in non-prophylaxis group. Our previous research also found that after August 2013 patients could receive more cycles of treatment due to medication assistance and health care policy.

In the majority of the cases, VZV reactivation occurred after finishing the second cycle of treatment, with or without antiviral prophylaxis. The onset of herpes zoster reported in the Phase III APEX Study took place during early treatment (31 days) (1). Thus, we should be particularly vigilant concerning the occurrence of VZV reactivation during the early bortezomib treatment period. On the other hand, herpes zoster can also be observed during the late stage of bortezomib treatment. Therefore, the prophylactic treatment should be continued throughout the whole course. No patient developed HZ after the cessation of bortezomib treatment, which may imply that it is unnecessary to prolong antiviral prophylaxis after stopping bortezomib therapy.

Due to the retrospective nature of this study, some detailed toxicity information was not available. In this study, we mainly focused on the safety profile including hepatic and renal toxicities. Abnormal liver function and renal dysfunction was observed in 12% and 2.8%, respectively, of the patients in the intermittent famciclovir group. These rates seemed to be higher than those in other reports, while the rates of the intermittent famciclovir group and the continuous acyclovir group were similar in our cohort. This might be related to the fact that we analyzed the toxic effect during the whole course of treatment. Bortezomib or other anti-myeloma drugs such as lenalidomide might have also contributed to the high occurrence of side effects we observed. Nonetheless, intermittent famciclovir prophylaxis was as safe as continuous acyclovir prophylaxis, even in patients with an increased serum creatinine level at baseline.

## Conclusion

It is necessary to use prophylaxis to prevent bortezomib-related herpes zoster infections in newly diagnosed multiple myeloma patients. Intermittent famciclovir prophylaxis is as efficient and safe as continuous acyclovir prophylaxis, yet can markedly reduce the duration and frequency of the administration of oral medicine, which may lead to better compliance and lighten the psychological burden for patients. Thus, it can be further applied to clinical practice.

## Data Availability Statement

The raw data supporting the conclusions of this article will be made available by the authors, without undue reservation.

## Ethics Statement

The studies involving human participants were reviewed and approved by the ethical committee of the First Affiliated Hospital, School of Medicine, Zhejiang University. The patients/participants provided their written informed consent to participate in this study.

## Author Contributions

GZ and ZC contributed to conception and design of the study. LY, JH, and EZ organized the database. FG and YZ performed the statistical analysis. FG and WW wrote the first draft of the manuscript. YY, XH, and DH wrote sections of the manuscript. All authors contributed to manuscript revision, read, and approved the submitted version.

## Funding

The study was financially supported by grants from Clinical research fund project of Zhejiang Medical Association.Program Number: 2018ZYC-A16.

## Conflict of Interest

The authors declare that the research was conducted in the absence of any commercial or financial relationships that could be construed as a potential conflict of interest.

## Publisher’s Note

All claims expressed in this article are solely those of the authors and do not necessarily represent those of their affiliated organizations, or those of the publisher, the editors and the reviewers. Any product that may be evaluated in this article, or claim that may be made by its manufacturer, is not guaranteed or endorsed by the publisher.
